# Suggestions as to the Method of Using Narcotics in Nervous Diseases

**Published:** 1855-07

**Authors:** John R. Allen

**Affiliations:** Prof. of Obstetrics and Diseases of Women and Children in Iowa Medical Department


### SUGGESTIONS AS TO THE METHOD OF USING NARCOTICS IN NERVOUS DISEASES.

BY JOHN R. ALLEN, M D.,

Prof. of Obstetrics and Diseases of Women and Children in Iowa Medical Department.

While the functions of the nerves have been elaborately investigated, and to a great extent satisfactorily established, their morbid states are comparatively little understood, and necessarily their treatment is more or less empirical and unsuccessful.

The various forms of neuroses apparently are becoming wonderfully prevalent, and I think especially so in our western cities. A large proportion of the ladies of families, who can afford to indulge in indolence and inactivity, and are confined to the heated and impure air of the town, and yet subject whenever they go out to the sudden vicissitudes and piercing winds of our climate, thus rendered in a high degree nervous and susceptible, are more or less troubled with some form of nervous disorder. To preserve the *complexion* the general system is subjected to habits, at war with the demands of nature, and the penalty is, a state of nervous excitibility which subjects them, in a great number of instances, to the tortures of neuralgia. Females of this class, are now too commonly taught to believe that there are but three epochs in life—to "come out," "find a husband," and "obtain an establishmement," but I will add, and *get—the neuralgia!*

The higher objects of life, the nobler duties of mother and citizen are forgotten, and the philosopher is now puzzled to determine which of the two evils is the the greater—the masculine effrontery of bloomerism and women's rights fanaticism, which at least tend to the more robust developement of the sex; or, the system which reduces to mere nervometers the children of the oppulent and indolent.

Formerly, when one of our grandmothers "caught a pain" by the great exposures which they were compelled to encounter, and which they did fearlessly, there was the stamen upon which to plant a lever of treatment, and her "rheumatics" surrounded to the vigorous measures which a firm constitution demanded. Now-a-days, however, these antiquated "rheumatics" have been refined by transmigration through the attenuated structure of your fine ladies, and uphoniously christened neuralgia, and do not demand or justify active treatment, and resort must be had to paliative and often empirical measures.

The difficulties which environ nervous pathology, it is true, are owing to the inscrutable nature of nervous function essentially considered. When we say we have discovered the function of a nerve, all we mean is, that the general office performed is known, not the occult processes by which the general result has been produced.

We are undoubtedly well acquainted with the origin, course, and termination of nerves, their special functions, and direct and reflex influences, but know little of the integral changes or essential action by which they are manifested.

Under the embarrassment into which we are thrown by the obscurity of disordered nervous action, it is scarcely to be wondered at, that we have been driven to the use of a class of remedies, as curative agents, of whose action we were equally ignorant, and where cures are effected, to attribute them rather to happy experiment, than to a priori determination of the applicability of the medicines used. Thus the salts of bismuth, of zinc, &c., have been used, sometimes successfully, but in a good number of cases the disease has proven incurable under every ordinary course of treatment.

We are aware of various pathological states which impair and even destroy nervous tissue, and others which destroy, impair or pervert nervous influence and function. We have atrophy, degenation, gangrene; we have also neuritis, neurilitis and neuralgia.

It is the last that we now wish to consider. We know there is a disorder of the nervous force inducing pain, spasm, &c., independant of any evidences of inflammation. We farther know, that this state may continue an indefinite period before indications of structural lesions occur. Instances of this kind of disease are very common.

Evidence of the production of pain, independently of any inflammatory action is constantly observed, in its location at a distance from the known point of irritation; thus, in diseased hip-joint, we find the knee complaining; the head from disturbed digestion, and the like. It is also true that we may have an *inflammatory element* imparted to the nervous force without the accompaniment of pain and this tendency developed at some point at a distance from the point of irritation, but usually in close relation with it by nervous connection, as where the retina of the closed eye of the microscopist becomes inflamed, in consequence of the over exertion of the one in use, and this may be extended to the conjunctiva, as observed by Paget more than once. Again, we see it in inflamed testicle from irritation of the urethra, and sometimes where the pain of a neuralgic attack is prolonged, indications of inflammation more or less intense succeed.

Now I have no intention of attempting an explanation of the difficulties by which this whole matter is surrounded, not only in a speculative, but a practical point of view. But to turn attention to a *mode of using* a class of agents, which are every day appealed to in the treatment of nervous diseases. This class of agents of course is narcotic.

I am very fully convinced that while we all regard these remedies as precious, nay invaluable agents, we have not given them that degree of consideration as *curative* which they deserve. We have much, nay almost all to learn, as to their value in this respect. We turn to them with alacrity and confidence in pain, and feel in moments of physical anguish that there is a relief, if not a cure at hand, and to no medicine do we award more directedness of action than these. Opium has been enthusiastically called "magnum dei donum," and to the body racked with pain, it may well so appear. But there is a very important aspect in which narcotics deserve much greater attention than they have recieved. Now in disorders which affect the quality of the blood or the functions of nutrition generally, which manifest themselves in general disturbance or local affections, whether of function or structure, we are very often called upon, not only to prescribe for present inconvenience and danger; but to endeavor, by a prolonged and gradual course of medication, to so change the state of the system, and to give to the vital energies a chance to re-establish their normal powers, and restore the physical integrity and functional activity Thus we often attempt a cure, and sometimes successfully, in neuroses, by means acting through the general system, by measures which do not act very sensible or directly on the nervous system. It is to an *alterant narcotic* course of medication which I desire to call attention.

Placed, professionally, for a number of years where I had ample opportunity of observing several forms of nervous disorders of the most serious class, such as insanity, epelpisy, &c., observing how frequently they existed unaccompanied with other signs of physical disorder than mental aberation in the one, convulsions, &c., in the other, I could not well see the indications of medication, unless something could be done, which was neither antiphlogistic nor yet repleting. It was but natural to turn to the class of remedies which we regarded as soothing to the nervous system. In cases of these forms of disease, I often found a confirmation of the statements of others, that they consisted of irritation, not of inflammation or congestion. True, the tendency of such condition was to inflammation and its results, but they were often primarily, but irritation, requiring an uncertain period to develope the inflammatory element.

Now in insanity we must allow the *mental excitement* to answer to the *pain in* neuroses, located in other portions of the system, and, if you please, the convulsions in epilepsy to correspond to the same. In the treatment of such cases, the object is to overcome the condition of the nervous system upon which these symptoms depend, and the means to do so we will assume as narcotics, prescribed in a certain way. True, some cases are reported cured by a single enormous dose of some narcotic, but these are exceptional, and the more rational mode seems to be that we would, by a gradual but persistent cause, overcome the habitual morbid preponderance and give the natural powers the opportunity to react.

We do not hope to cure anæmia by a single dose of iron, nor a debility of the stomach by a few tonic potions. Indeed we attempt to avoid as much as possible any sensible or immediate effect and to rely upon that slow, but permanent influence by which the blood is enriched and the tone of the system is restored for the cure of these disorders.

These remedies are durable agents in the blood or tissues, are used to counteract the causes of prolonged and inveterate diseases, and make a lasting impression on the system. When remedies of this class are used, we understand to some extent, the nature of their action, it often being comparable to known chemical processes, and thereby meeting a chemical deficiency, so to express it, which we previously believed to exist in the system. But as we have seen that the changes of the system in nervous diseases, whatever they may be, are often altogether far beyond our apprehension, so the action of neurotics are also equally inexplicable.

Diseases of a nervous character of very different origin, or rather occurring in states of the system very unlike each other, may present very similar features. Thus we may have very high nervous excitement from a state of plethora, and again from vascular exhaustion. So also very different symptoms may arise from the same apparent seat and cause of disease.

We may have a high degree of maniacal exhaltation, or a condition of torpid stupidity from cerebral irritation. We may have a paralysis or a neuralgia from apparently similar cause; and, we may have any of these manifestations for an uncertain period, and no appreciable cause to which to refer them, and no lesion commensurative with their symptoms. While these maladies are thus clothed in mystery on the other hand, the same inscrutable secrecy surrounds the modus operandi of the class of remedies known as neurotics. We know little of the ultimate causes of sensation, motion, or nervous excitement, and we cannot know much of the action of agents which affect them.

We find that they produced no change in the blood, do not remain in it, but soon pass out. They seem to act upon nerve fibre by actual contact, but soon cease to act. Their action is transitory, though often powerful. Chemistry supplies us no explanation, and among the best of modern writers gives, as the most plausible conjectural account of their action, "the mechanical theory." We have, at all events, as will be percieved, a sort of homœopathic similtude in the inscrutable nature of the disease, and the inexplicable action of the remedies proposed for their relief.

The effects, however, both of the diseases and remedies are very palpable. The more important symptoms, are those which affect the intellect, sensation and motion. The effects on mind of disordered nervous matter is observed in the varieties of insanity, of sensation in the pains of neuralgia, of motion in convulsions.

